# Study on the Structure and Antioxidant Properties of Seamless Knitted Fabrics with Antioxidant Fibers

**DOI:** 10.3390/ma18235446

**Published:** 2025-12-03

**Authors:** Lei Yan, Lu Chang, Shuhan Shen, Zimin Jin, Mingtao Zhao

**Affiliations:** 1 College of Textile Science and Engineering (International Silk College), Zhejiang Sci-Tech University, Hangzhou 310018, China; yan_lei2001@163.com (L.Y.); ssh_aa123@163.com (S.S.); 2 Shanghai Yijin Testing Technology Co., Ltd., Shanghai 200001, China; cherry@test-china.com; 3 Zhejiang Bangjie Holding Group Co., Ltd., Yiwu 322000, China; mtzhao@bangjie.cn

**Keywords:** antioxidant fiber, seamless knitting, free radicals, antioxidant

## Abstract

The antioxidant properties of seamless knitted fabrics with antioxidant fibers determine their antioxidant effects. In this paper, we used five kinds of raw yarn materials, namely mint nylon filament, tea carbon nylon filament, coffee carbon nylon filament, collagen nylon filament, and nylon filament, and chose three kinds of fabric microstructure, namely weft flat knit, 1+1 false rib, and 1+3 false rib, to obtain 15 seamless knitted samples with antioxidant fiber by establishing the sample scheme through the full factorial experimental method and weaving them on the seamless loom. DPPH and ABTS free radical scavenging tests were performed on the 15 seamless knitted samples of antioxidant fiber according to the standards, and the results showed that, in terms of the type of yarns, the antioxidant performance of tea carbon nylon filament was the best, followed by coffee carbon nylon filament and mint nylon filament, and the antioxidant performances of collagen nylon and ordinary nylon yarn were relatively weak; in terms of the fabric structure, the 1+1 false rib structure was slightly better than the weft flat knit structure, while the 1+3 false rib structure was relatively poor. Overall, the antioxidant performance of sample No. 5, with the 1+1 false rib structure and tea carbon nylon thread, was the best.

## 1. Introduction

Antioxidant functional textiles, which are textiles with antioxidant active ingredients that exert antioxidant skincare effects when in contact with the skin, have received increasing attention from researchers and consumers in recent years. Accordingly, antioxidant function has become a new exploratory field in the development of functional textiles [1].

Antioxidants are a class of substances that can delay or inhibit oxidative reactions by scavenging free radicals, blocking oxidative chain reactions, or repairing oxidative damage [2]. Based on their mechanism of action and source, antioxidants can be divided into two broad categories, which can be classified as enzymatic antioxidants and non-enzymatic antioxidants [3]. Enzymatic antioxidants are endogenous oxidants that act by directly scavenging ROS [4], such as superoxide dismutase, catalase, and other peroxidases. Non-enzymatic antioxidants are exogenous oxidants that assist the first group of antioxidant enzymes and perform other tasks. For example, vitamins block lipid peroxidation [5], and polyphenols can scavenge free radicals through phenolic hydroxyl groups [6].

The main functional component in mint nylon filament is mint essential oil. Xian et al. [7] extracted mint essential oil using an ultrasonic-assisted method and found that it exhibits good antioxidant activity in vitro. When the concentration of mint essential oil was 0.9 mg/mL, its ability to scavenge hydroxyl radicals reached 75.00%. Luteolin and hesperidin in mint can provide hydrogen atoms through the ortho-diphenol hydroxyl groups of ring B to scavenge free radicals and activate the Nrf_2_ pathway to enhance the intrinsic antioxidant capacity of cells [8]. Zhao et al. [9] used luteolin to functionalize silk and found that silk dyed directly with luteolin showed a significant improvement in the scavenging rates of ABTS and DPPH compared to untreated silk, indicating that its attachment to silk imparts certain antioxidant properties to the fabric.

Tea carbon nylon filament is a functional yarn made by processing tea into a carbon material with a microporous structure through a series of treatments, then composite-spinning it with nylon. The functional components in tea, tea polyphenols, are mainly composed of catechins, flavonoids, anthocyanins, and phenolic acids, among which catechins account for 60–80% of tea polyphenols [10], and epicatechin is one of the most common isomers of catechins. Lei et al. [11] modified epicatechin in tea through carbonylation modification and found that the modified epicatechin significantly improved its ability to scavenge ABTS, DPPH, and O^2−^ radicals.

Coffee carbon fiber is a new type of environmentally friendly fiber produced by calcining waste coffee grounds at temperatures above 1000 °C, micronizing them using the latest nanotechnology, and adding them to synthetic fibers [12]. Coffee grounds contain phenolic acid components such as chlorogenic acid, caffeic acid, ferulic acid, p-coumaric acid, gallic acid, and protocatechuic acid. Chlorogenic acid can provide electrons or hydrogen atoms to eliminate reactive oxygen radicals, and caffeic acid neutralizes lipid peroxyl radicals through the synergistic action of carboxyl and hydroxyl groups and inhibits tyrosinase activity to reduce melanin oxidation deposition [13]. Hong et al. [14] extracted coffee grounds using a manual espresso machine and used tannic acid as a mordant to dye wool fabrics, significantly improving the light fastness and antibacterial rate of the fabrics. Combining coffee grounds with fabrics endows fabrics with antioxidant, UV protection, and antibacterial functional properties.

Collagen plays a key role in maintaining the function and morphology of the skin [15], and a reduction in collagen content is considered an important indicator of skin aging [16]. After extracting collagen solution from the skin of deep-sea fish, collagen molecules are mixed with wood pulp fibers using nanotechnology and then processed through spinning and other processes to produce collagen yarn. Collagen fibers have a soft texture, good hygroscopicity, high moisture regain rate, excellent moisture retention and breathability, and good affinity with the skin [17].

This study aims to endow fabrics with the potential to protect human skin from oxidative stress damage by combining different antioxidant fibers with seamless knitting technology and to evaluate and compare the antioxidant capacity of the fabrics. In addition, these yarns also have antibacterial, UV-resistant, and improved color fastness properties. It is hoped that this study can provide a certain theoretical basis and experimental foundation for the development of multifunctional textiles.

## 2. Materials and Methods

### 2.1. Materials

#### 2.1.1. Selection of Yarn Program

This thesis focuses on exploring the mechanism of the influence of different antioxidant fibers on the skin protection performance of seamless knitted fabrics. In the raw material selection of yarn, to avoid the influence of fiber fineness on fabric performance, four functional yarns of 7.78 tex (70 Denier/48 Filaments, 70 D/48 F) specifications were uniformly selected, which were mint nylon yarn, tea carbon nylon yarn, coffee carbon nylon yarn, and collagen nylon yarn, and nylon yarn samples of the same specifications were set up as the control samples in order to ensure the comparability of the experimental data. 22.2 dtex (20 D)/22.2 dtex (20 D) nylon/spandex-covered yarn was used as the lining yarn to enhance the handfeel and comfort of the fabric. The specific raw yarn materials and specifications are shown in Table 1.

#### 2.1.2. Fabric Structure Design

**Figure 1:**
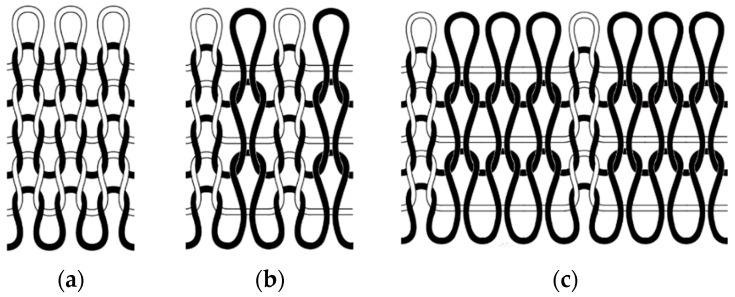
Fabric structures: (**a**) weft flat knit; (**b**) 1+1 false rib; (**c**) 1+3 false rib.

For the design, we selected the weft flat knit, 1+1 false rib, and 1+3 false rib structures, three commonly used seamless knitted fabric microstructures. Coil diagrams of the three structures are shown in Figure 1.

(1) Weft flat knit

As one of the most common fabrics in daily life, the good elasticity and extensibility of flat knit fabrics can make them more flattering and allow them to adapt to various postures of the human body, so the flat knit is widely used [18]. And because flat knit is relatively simple, it is easy to produce [19].

(2) 1+1 false rib

1+1 false rib is a kind of improved double-sided weft knitting structure, whose structural characteristic is that the longitudinal rows of the front side loops and the longitudinal rows of the reverse side loops are arranged alternately in a ratio of 1:1. This structure has good elasticity and extensibility when stretched horizontally, enabling the fabric to maintain a snug and comfortable fit. The ribbed structure is symmetrical and stable, and its fabrics thus obtain a more stable appearance and better durability [20].

(3) 1+3 false rib

1+3 false rib, as a variation of the false rib series, shows an obvious ribbing effect on the front side and a regular long floating line structure on the back side. Its fabric tightness is reduced by 15–20% compared with 1+1 false rib. The transverse extensibility is improved by 30–40%, which makes it more suitable for application scenarios requiring larger deformation [21].

#### 2.1.3. Establishment of Specimens

This study adopts a two-factor full factorial experimental design method to systematically investigate the interactive effects of yarn type and fabric structure on fabric functional properties. The experimental design contained two key variables: yarn type (Factor A) consisted of five levels, namely mint nylon filament, tea carbon nylon filament, coffee carbon nylon filament, collagen nylon filament, and nylon filament, and the fabric microstructure (Factor B) was set up with three levels, namely weft flat knit, 1+1 false rib, and 1+3 false rib. A table of factor levels is shown in Table 2. The detailed specific specimen program is shown in Table 3.

### 2.2. Experiments and Tests

In living organisms, the generation and scavenging of free radicals maintain a sophisticated system of dynamic equilibrium. This equilibrium is essential for maintaining normal physiological functions, but when the organism is subjected to external environmental stimuli or internal metabolic abnormalities, the rate of free radical production may significantly exceed the body’s scavenging capacity, leading to a state of oxidative stress.

In this study, the scavenging effects of DPPH and ABTS radicals were investigated using 15 samples to investigate their antioxidant properties, to explore the effects of different yarn types and organizational structures on the antioxidant effects of fabrics, and to provide an important basis for the production of antioxidant garments.

Experimental materials: Fifteen fabric specimens were prepared. Each specimen (cut into pieces of approximately 5 mm × 5 mm) had a total mass of no less than 12 g. From each specimen, a 2 g portion (accurate to 10 mg) was taken for experimentation.

Experimental reagents: laboratory-made deionized water, anhydrous ethanol (purity ≥ 99.8%), 2,2-diphenyl-1-picrylhydrazyl (DPPH), and potassium persulfate were all analytical-grade reagents purchased from Shanghai Aladdin Bio-Chem Technology Co., Ltd. (Shanghai, China); 2,2′-azino-bis(3-ethylbenzothiazoline-6-sulfonic acid) diammonium salt (ABTS) was an analytical-grade reagent purchased from Tianjin Zhonglian Chemical Reagent Co., Ltd. (Tianjin, China).

Experimental equipment: FA2004 analytical balance, Shanghai Hengping Scientific Instrument Co., Ltd. (Shanghai, China), and UV-3000BPC ultraviolet–visible spectrophotometer, Shanghai Meipuda Instrument Co., Ltd. (Shanghai, China).

The free radical scavenging rate is calculated as Equation (1).

(1) $$P=(1-\frac{A_{1}-A_{2}}{A_{0}})\times 100\%$$

where *P* is the free radical scavenging rate, %; *A*_1_ is the absorbance of the specimen mixed with DPPH solution/ABTS solution; *A*_2_ is the absorbance of the specimen mixed with anhydrous ethanol/deionized water; and *A*_0_ is the absorbance of the DPPH solution/ABTS solution.

#### 2.2.1. DPPH Radical Scavenging Rate Test Experiment

Experimental reference: In this study, we refer to the standard “T/CCTA 20102-2023 Determination and Evaluation of Textile Antioxidant Capacity by DPPH and ABTS Method” [22] and use the DPPH and ABTS methods to determine the antioxidant performance of fabrics. In the actual testing process, due to the different sensitivities of the UV–visible spectrophotometer, the concentration of DPPH solution was increased to ensure that the concentration of DPPH solution was within the range of absorbance 0.7 ± 0.02 to ensure the accuracy and reliability of the test results.

Reagent preparation: A total of 10 mg of DPPH was accurately weighed, dissolved in 250 mL of anhydrous ethanol, and configured into a 40 mg/mL DPPH solution, which was ready for use.

**Figure 2:**
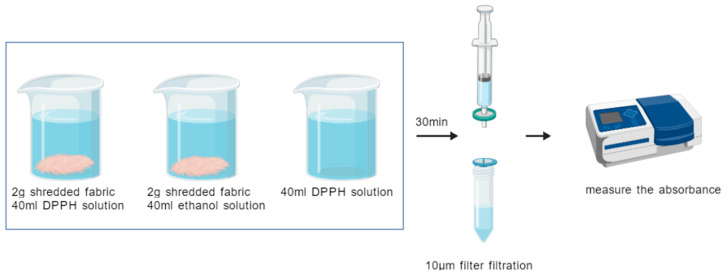
Schematic diagram of the experimental procedure for DPPH radical scavenging.

Experimental method: First, 2 g of sheared fabric was placed in beaker 1 and 40 mL of DPPH solution was added. Control group 1 involved placing an equal volume of ethanol (as a substitute for DPPH solution) into beaker 2. And control group 2 took 40 mL of DPPH solution, which was placed in beaker 3. The contents of the three beakers were mixed well and left at room temperature for 30 min before being filtered through a 10 μm organic system needle filter, and the filtrate was collected for testing. The filtrate was used to determine the absorbance at 517 nm by a UV–visible spectrophotometer and zeroed using anhydrous ethanol. The specific experimental operation is shown in Figure 2.

#### 2.2.2. ABTS Radical Scavenging Rate Test Experiment

Experimental reference: This study refers to the standard “T/CCTA 20102-2023 Textiles Determination and Evaluation of Antioxidant Ability DPPH and ABTS Method”.

Reagent preparation: First, 200 mg of ABTS and 34.4 mg of potassium persulfate were weighed, dissolved in 50 mL of deionized water, shaken well, and then placed at room temperature and protected from light for 24 h. Then, an appropriate amount of ABTS was taken and diluted with 95% ethanol until the absorbance was within the range of 0.7 ± 0.02. In this study, 20 mL of ABTS solution was matched with about 700 mL of 95% ethanol solution, as ABTS working solution, which was ready for use.

**Figure 3:**
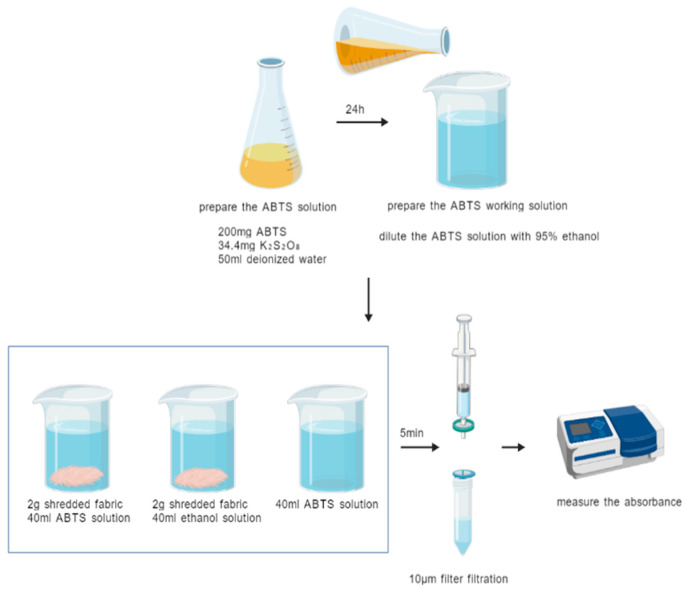
Schematic diagram of the experimental procedure for ABTS radical scavenging.

Experimental method: First, 2 g of sheared fabric was placed in beaker 1 and 40 mL of ABTS working solution was added, while for control group 1, an equal volume of deionized water was taken instead of the mixed solution and placed in beaker 2, and for control group 2, 40 mL of ABTS working solution was taken and placed in beaker 3. The contents of the three beakers were mixed well and left at room temperature for 5 min before being filtered through a 10 μm organic system needle filter, and the filtrate was collected for testing. The filtrate was used to determine the absorbance at 734 nm using a UV–visible spectrophotometer and zeroed using deionized water. The specific experimental operation is shown in Figure 3.

## 3. Results and Discussions

### 3.1. DPPH Radical Scavenging Rate Test Results

The DPPH radical scavenging rate test results of 15 specimens were obtained according to the above experimental method test, as shown in Table 4.

The results from Table 4 were analyzed and the test data were tested by SPSS 27.0 for a normal distribution. Furthermore, a two-way analysis of variance [23] was used to study the correlation between the DPPH radical scavenging rate of 15 samples and their veil types and fabric structures, and the results of the between-subjects effect test are presented in Table 5.

From the results of the between-subjects effect test, it can be seen that the significance *p*-value of factor A, raw yarn material, is less than 0.001, indicating that yarn type has an extremely significant effect on the DPPH radical scavenging rate of the fabrics. The significance *p*-value for the factor B fabric structure is 0.169, indicating that the effect of the fabric microstructure on fabric DPPH radical scavenging is less significant.

**Figure 4:**
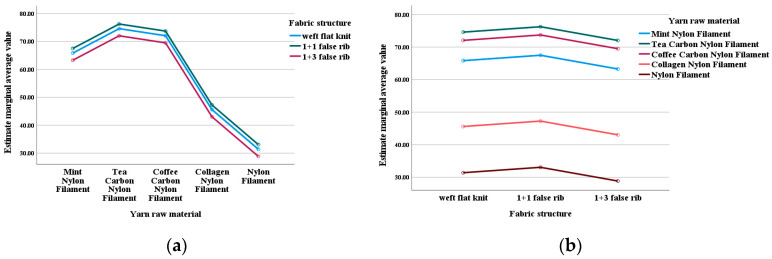
Estimated marginal mean fabric DPPH free radical scavenging rate. (**a**) Dependent variable is raw yarn material. (**b**) Dependent variable is fabric structure.

The estimated marginal means presented in Figure 4 provide a more intuitive numerical comparison.

In terms of yarn types, tea carbon nylon exhibits the highest DPPH free radical scavenging rate, followed by coffee carbon nylon and mint nylon, while collagen nylon and ordinary nylon are relatively lower. From the numerical trend, tea carbon nylon demonstrates the most excellent antioxidant performance, indicating that the antioxidant performance of fabrics is closely related to the content of polyphenols and flavonoids. During subsequent extraction and content determination of polyphenols and flavonoids in the three types of yarns, it was found that the tea carbon nylon yarn had the highest content of polyphenols and flavonoids, followed by the coffee carbon nylon yarn, and the mint nylon yarn had the lowest. Additionally, the content of collagen in collagen yarn was much lower than that in the other three types of yarn, so the effect was not ideal.

In terms of fabric structure, the DPPH scavenging rate of 1+1 false rib was slightly higher than that of weft flat knit, while that of 1+3 false rib was relatively lower. The 1+1 false rib structure is more conducive to the exertion of antioxidant activity because it is balanced and stable, with uniform stress distribution [24]. When subjected to shear, it can produce a large number of uniformly sized small fragments, thereby maximizing the effective specific surface area, allowing for the most thorough contact with the solvent and dissolution of antioxidant components. The weft flat knit structure is simple but unstable, resulting in moderate dissolution effects. The 1+3 false rib structure is highly unbalanced, which slows down the overall dissolution rate, leading to the worst performance.

### 3.2. ABTS Radical Scavenging Rate Test Results

The ABTS radical scavenging rate test results of the 15 specimens obtained according to the above experimental method test are shown in Table 6.

From the results in Table 6, it can be seen that the significance of ABTS’s free radical scavenging rate compared to DPPH increased, which may be due to the fact that the ABTS method only requires a rapid reaction of 5 min. Furthermore, a two-way analysis of variance was used to study the correlation between the ABTS radical scavenging rate of the 15 samples and their veil types and fabric structures, and the results of the between-subjects effect test are presented in Table 7.

From the results of the between-subjects effect test, it can be seen that the significance *p*-value of factor A, raw yarn material, is less than 0.001, indicating that yarn type has a highly significant effect on the fabric ABTS radical scavenging rate. The significance *p*-value for the factor B fabric microstructure is 0.082, indicating that the effect of tissue structure on the fabric ABTS radical scavenging rate is less significant.

**Figure 5:**
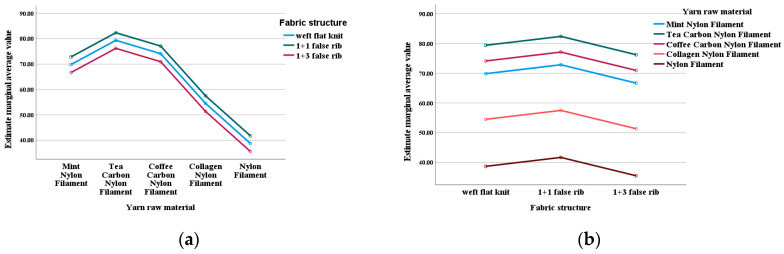
Estimated marginal mean of fabric ABTS free radical scavenging rate. (**a**) Dependent variable is raw yarn material. (**b**) Dependent variable is fabric structure.

The estimated marginal means presented in Figure 5 provide a more intuitive numerical comparison.

Regarding yarn types, tea carbon nylon shows the highest ABTS free radical scavenging rate, followed by coffee carbon nylon and mint nylon; collagen nylon and ordinary nylon have relatively lower rates. Numerical trends indicate that tea carbon nylon has the best antioxidant performance, suggesting that fabric antioxidant properties are closely linked to polyphenol and flavonoid content. Further analysis of polyphenol and flavonoid content in three yarn types revealed that tea carbon nylon yarn has the highest levels, followed by coffee carbon nylon yarn, with mint nylon yarn having the lowest. Additionally, collagen yarn contains far less collagen than the other three yarn types, resulting in less effective performance.

Regarding fabric structure, the ABTS scavenging rate of 1+1 false rib is slightly higher than weft flat knit, while 1+3 false rib has a relatively lower rate. The 1+1 false rib structure enhances antioxidant activity due to its balanced and stable nature with a uniform stress distribution. Under shear stress, it produces numerous uniformly sized small fragments, maximizing the effective specific surface area and enabling thorough contact with solvents for optimal dissolution of antioxidant components. Weft flat knit structures, though simple, are unstable, leading to moderate dissolution effects. The 1+3 false rib structure is highly unbalanced, slowing overall dissolution and resulting in the poorest performance.

## 4. Conclusions

In this study, the DPPH radical scavenging rate and ABTS radical scavenging rate of 15 seamless knitted specimens with antioxidant fibers were tested, and the experimental data were analyzed as follows:

(1) The DPPH and ABTS free radical scavenging rate results of 15 samples were analyzed. Both showed that, for yarn types, the free radical scavenging rates arranged from smallest to largest followed the order nylon filament < collagen nylon filament < mint nylon filament < coffee carbon nylon filament < tea carbon nylon filament, while for fabric structure, the free radical scavenging rates arranged from smallest to largest followed the order 1+3 false rib < weft flat knit < 1+1 false rib.
(2) In the antioxidant performance tests of both DPPH radical scavenging experiments and ABTS radical scavenging, specimen #5 using tea carbon nylon filament as raw yarn material with a fabric structure of 1+1 false rib had the best antioxidant performance of all types. However, the antioxidant performance of specimen #15 with the 1+3 false rib structure using nylon filament as a coating was poor.
(3) This study confirms that knitted fabrics made from tea carbon nylon yarn with a 1+1 false rib structure possess excellent antioxidant properties. These fabrics show great potential for subsequent applications. First, they could be used to develop high-end intimate apparel that combines comfort and antibacterial functions. Second, they are suitable for manufacturing healthy home textiles and baby–mother textiles. Third, they also have application prospects in industrial textiles such as medical protection. This work provides a new direction and material basis for the development of high-value functional textiles and the high-value utilization of tea resources.

## Figures and Tables

**Table 1:** Raw yarn materials and specifications.

Raw Yarn Materials	Fineness	Yarn Name	Supplier
Yarn	7.78 tex (70 D/48 F)	Mint Nylon Filament	Shandong Rare Technology Co. (Jinan, China)
		Tea Carbon Nylon Filament	Chinachem New Material Technology Co. (Xiamen, China)
		Coffee Carbon Nylon Filament	Chinachem New Material Technology Co. (Xiamen, China)
		Collagen Nylon Filament	Taicang Fangke Textile Co. (Suzhou, China)
		Nylon Filament	Yiwu Huading Nylon Co. (Jinhua, China)

**Table 2:** Factor level.

No.	Factor	
	A (Raw Yarn Materials)	B (Fabric Microstructures)
1	Mint Nylon Yarn	weft flat knit
2	Tea Carbon Nylon Yarn	1+1 false rib
3	Coffee Carbon Nylon Yarn	1+3 false rib
4	Collagen Nylon Yarn	---
5	Nylon yarn	---

**Table 3:** Fabric sample program.

Specimen No.	A (Raw Yarn Materials)	B (Fabric Microstructures)
#1	Mint Nylon Yarn	weft flat knit
#2	Mint Nylon Yarn	1+1 false rib
#3	Mint Nylon Yarn	1+3 false rib
#4	Tea Carbon Nylon Yarn	weft flat knit
#5	Tea Carbon Nylon Yarn	1+1 false rib
#6	Tea Carbon Nylon Yarn	1+3 false rib
#7	Coffee Carbon Nylon Yarn	weft flat knit
#8	Coffee Carbon Nylon Yarn	1+1 false rib
#9	Coffee Carbon Nylon Yarn	1+3 false rib
#10	Collagen Nylon Yarn	weft flat knit
#11	Collagen Nylon Yarn	1+1 false rib
#12	Collagen Nylon Yarn	1+3 false rib
#13	Nylon Yarn	weft flat knit
#14	Nylon yarn	1+1 false rib
#15	Nylon Yarn	1+3 false rib

**Table 4:** Test results of DPPH radical scavenging rate of fabrics.

Specimen No.	DPPH Radical Scavenging Rate/%	Specimen No.	DPPH Radical Scavenging Rate/%
#1	64.6	#9	68.1
#2	68.9	#10	45.2
#3	63.1	#11	43.0
#4	75.0	#12	47.7
#5	79.9	#13	30.7
#6	68.0	#14	32.8
#7	74.0	#15	29.8
#8	73.2		

**Table 5:** Between-subjects effect test for DPPH radical scavenging rate of fabrics.

Dependent Variable: DPPH Radical Scavenging Rate					
Source	Class III Sum of Squares	Degrees of Freedom	Mean Square	F	Significance
Modified model	4233.383 ^a^	6	705.564	69.895	<0.001
Intercept	49,766.400	1	49,766.400	4929.970	<0.001
A (raw yarn material)	4188.187	4	1047.047	103.723	<0.001
B (fabric microstructure)	45.196	2	22.598	2.239	0.169
Error	80.757	8	10.095		
Total	54,080.540	15			
Revised total	4314.140	14			

^a^ R-square = 0.981 (adjusted R-square = 0.967).

**Table 6:** Test results of ABTS radical scavenging rate of fabrics.

Specimen No.	ABTS Radical Scavenging Rate/%	Specimen No.	ABTS Radical Scavenging Rate/%
#1	71.4	#9	70.0
#2	72.3	#10	48.3
#3	65.7	#11	58.2
#4	80.4	#12	56.9
#5	85.1	#13	39.7
#6	72.5	#14	40.6
#7	76.8	#15	35.7
#8	75.4		

**Table 7:** Between-subjects effect test for ABTS radical scavenging rate of fabrics.

Dependent Variable: ABTS Radical Scavenging Rate					
Source	Class III Sum of Squares	Degrees of Freedom	Mean Square	F	Significance
Modified model	3395.072 ^a^	6	565.845	41.491	<0.001
Intercept	60,040.067	1	60,040.067	4402.518	<0.001
A (raw yarn material)	3300.187	4	825.047	60.498	<0.001
B (fabric microstructure)	94.885	2	47.443	3.479	0.082
Error	109.101	8	13.638		
Total	63,544.240	15			
Revised total	3504.173	14			

^a^ R-square = 0.969 (adjusted R-square = 0.946).

## Data Availability

The original contributions presented in this study are included in the article. Further inquiries can be directed to the corresponding author.

## References

[1] Zhang Y., Zhou Q., Rather L.J., Li Q. (2021). Agricultural waste of *Eriobotrya japonica* L. (Loquat) seeds and flora leaves as source of natural dye and bio-mordant for colouration and bio-functional finishing of wool Textile. Ind. Crops Prod..

[2] Li R., Pan X., Wang J., Yang J., Zhang J. (2020). Application and development of different kinds of antioxidants. Chem. Eng. Equip..

[3] Li S., Ma Y., Wang Y., Si W., Zhu C., Zhang H. (2024). Research progress of antioxidant textiles. Dye. Finish. Technol..

[4] Wang H., Yin X., Xia B., Zheng Q., Cui M. (2023). Protective Effect and Mechanism of Endogenous Antioxidant Metallothionein on Myocardial Damage Induced by Chronic Intermittent Hypoxia. Chin. J. Health Preserv..

[5] Zhu H.Y., He Y.M., Li M., Yang L. (2021). Analysis of in vivo lipid peroxidation status on AUB in dysfunctional AUB and AUB in organic diseases. Heilongjiang Med..

[6] Zhang J., Gao B., Ye B., Sun Z., Qian Z., Yu L., Bi Y., Ma L., Ding Y., Du Y. (2023). Mitochondrial—Targeted Delivery of Polyphenol—Mediated Antioxidases Complexes against Pyroptosis and Inflammatory Diseases. Adv. Mater..

[7] Xian K., Luo H., Li R., Wang H., Zhang J., Wang G.R. (2023). Extraction and Analysis of Antioxidant Activity of Menthol Essential Oil. Agric. Sci. Technol. Inf..

[8] Lai D., Zhu X., Li T., Zhang Q., Peng Q.H. (2023). Effect of Heating Methods on Nutritional Components and Antioxidant Activity of Purple Potato. Food Sci..

[9] Zhao P., Fang J., Zhao Y., Wang Y.N., Chen X., Cao H.M. (2024). Dyeing and Functional Modification of Silk with Curcumin and Luteolin. Dye. Finish..

[10] Wang Y. (2022). Study on Extraction of Tea Polyphenols from Green Tea by Pulsed Electric Field Combined with Ultrasonic Wave and Preparation of Instant Tea Powder. Master’s Thesis.

[11] Lei X., Zhang M., Lin H., Wang L.L., Zheng D.Y. (2024). Carbonylation Modification of Epigallocatechin, Its UV Absorption and Antioxidant Activity. J. Tea Sci..

[12] Ji L. (2022). Production Practice of Coffee Carbon Odor-Removing Functional Weft-Knitted Fabrics. Knitt. Ind..

[13] Pan C., Shi Y., Luo X., Ye T.S., Huang J.Y., Jiang T. (2025). Research Progress on the Role of Caffeic Acid and Its Derivatives in Diabetes and Its Complications. Cent. South Pharm..

[14] Hong K.H. (2018). Effects of Tannin Mordanting on Coloring and Functionalities of Wool Fabrics Dyed with Spent Coffee Grounds. Fash. Text..

[15] Li W. (2024). Preparation and Application Research of High-Strength Collagen Fiber. Master’s thesis.

[16] Hu H., Zhang H., Wang J., Wang L.W., Liu Q. (2025). Application of Recombinant Collagen in Biomedicine. Prog. Biochem. Biophys..

[17] Wang Q. (2023). Study on the Relationship between Structure and Properties of Seamless Knitted Fabrics of Protein Fibers for Autumn and Winter. Master’s Thesis.

[18] Mikučionienė D., Čiukas R., Mickevičienė A. (2010). The influence of knitting structure on mechanical properties of weft knitted fabrics. Mater. Sci..

[19] Chen W., Jin Z., Chen S., Fang C., Zheng C. (2024). Study on the Permeability and Absorption Performance of the Crotch Layer in Seamless Knitted Period Underwear. Materials.

[20] Kodžoman D., Hladnik A., Čuden A.P., Čok V. (2023). Assessment and semantic categorization of fabric visual texture preferences. Autex Res. J..

[21] Chen W., Jin Z., Mao L. (2025). Structure and Wear Comfort Performance of Aloe Vera Fiber Seamless Knitted Fabrics. Fash. Des. Eng..

[22] (2023). Determination and Evaluation of Oxidation Resistance of Textiles by DPPH and ABTS Methods.

[23] Min F., Hu Y., Hu D. (2024). Knowledge Understanding of Two-Factor Variance Analysis with Interaction in Probability and Statistics. Math. Commun..

[24] Wang Q., Lu J., Jin Z., Chen K., Zhao M., Sun Y. (2022). Study on the Structure and Skin Moisturizing Properties of Hyaluronic Acid Viscose Fiber Seamless Knitted Fabric for Autumn and Winter. Materials.

